# Multidrug-Resistant Shigellosis as a Sexually Transmitted Infection in Advanced HIV: A Case Report

**DOI:** 10.7759/cureus.64234

**Published:** 2024-07-10

**Authors:** Mannat K Bhatia, Fatima Dastagir, Abdul M Khan, Henry Redel

**Affiliations:** 1 Department of Internal Medicine, Rutgers Robert Wood Johnson Medical School, Saint Peter's University Hospital, New Brunswick, USA; 2 Department of Internal Medicine, St. George's University School of Medicine, Saint Peter's University Hospital, New Brunswick, USA; 3 Department of Infectious Diseases, Rutgers Robert Wood Johnson Medical School, Saint Peter's University Hospital, New Brunswick, USA

**Keywords:** shigellosis, advanced hiv disease, multidrug-resistance, men who have sex with men, healthcare transition, antimicrobial resistance‎

## Abstract

Shigellosis, a significant public health concern, has increasingly been recognized as a sexually transmitted infection (STI) among men who have sex with men (MSM), particularly in those with HIV. This case report describes a 25-year-old MSM with advanced HIV who presented with recurrent multidrug-resistant (MDR) *Shigella flexneri* infection despite multiple hospitalizations and antibiotic courses. The patient's high-risk sexual behaviors and suboptimal HIV management likely contributed to recurrent exposure to *Shigella* and the selection of resistant strains. This case highlights the complex interplay of individual behaviors, immune suppression, antimicrobial resistance, and the healthcare system in the context of this emerging STI. It underscores the importance of optimized HIV care, comprehensive patient education, robust healthcare coordination, and strengthened surveillance to effectively combat MDR shigellosis in vulnerable populations.

## Introduction

Shigellosis, an acute diarrheal illness predominantly caused by *Shigella sonnei* (81%) and *Shigella flexneri* (12.6%) [1], is a significant public health concern. According to the latest data from the Centers for Disease Control (CDC), an estimated 450,000 cases occur annually in the United States alone. Alarmingly, approximately 242,000 of these cases are multidrug-resistant (MDR) strains, posing a growing challenge for effective treatment.

Initially recognized as a disease primarily transmitted through the fecal-oral route, shigellosis has emerged as a sexually transmitted enteric infection among men who have sex with men (MSM), particularly in high-income countries. Since the first reported cases of MSM with HIV in the 1970s, international outbreaks of both *S. sonnei* and *S. flexneri* have been documented across major cities globally. The evolving drug resistance patterns among these strains further amplify the public health threat [2-4].

This case report presents a unique instance of recurrent MDR shigellosis in a young man with advanced HIV who engages in high-risk sexual behaviors. This case illustrates the complex convergence of individual behaviors, compromised immunity, evolving antimicrobial resistance (AMR), and healthcare system challenges in the context of sexually transmitted shigellosis.

## Case presentation

A 25-year-old college student presented to the emergency department in December 2022 with a four-day history of sharp, crampy lower abdominal pain, fever, chills, sweating, and multiple episodes of large-volume, watery diarrhea. This presentation was concerningly familiar, as he had been hospitalized twice earlier in the year for similar symptoms. In August, he was diagnosed with *S. flexneri* infection and HIV. Despite receiving a course of ciprofloxacin for the shigellosis, he did not follow up for HIV care and experienced a relapse in September. This time, the *Shigella *strain was resistant to multiple antibiotics, including ciprofloxacin. Although treated with meropenem, his HIV was not optimally managed (Truvada instead of Biktarvy was used), and his diarrhea never completely resolved.

**Figure 1:**
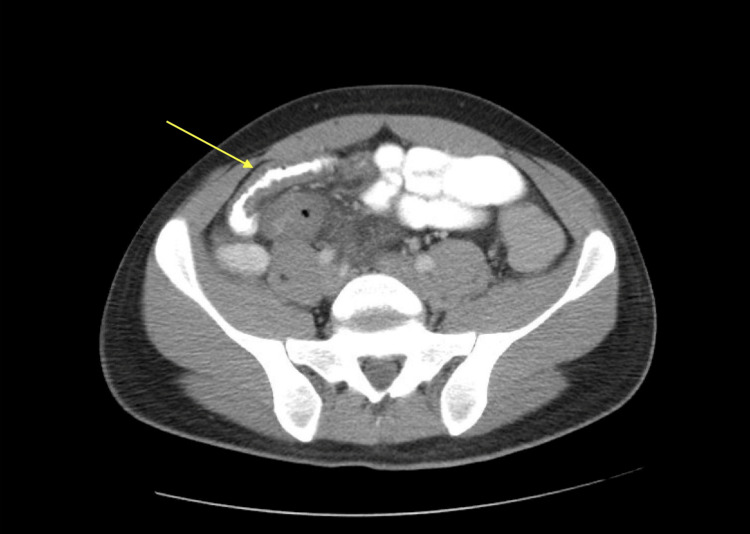
CT scan of the abdomen and pelvis with and without contrast The pointed arrow represents inflammation of the terminal ileum and segment of the distal ileum with prominent wall thickening and mural edema.

During this third hospitalization in February 2023, the patient reported ongoing high-risk sexual behaviors with multiple male partners. Examination revealed fever, tachycardia, and diffuse abdominal tenderness. Stool cultures confirmed MDR *S. flexneri*. His CD4 count had improved to 170 cells/μL (from 46 in September), but his HIV viral load remained high at 67,200 copies/mL. An abdominal CT scan showed acute enterocolitis (Figure 1).

Recognizing the need for comprehensive management, the patient was treated with intravenous ertapenem for shigellosis and switched to the recommended Biktarvy regimen for HIV, along with Bactrim for prophylaxis. This multifaceted approach led to the resolution of his symptoms, and he was discharged with a plan for close follow-up with the infectious disease (ID) clinic and continued oral ertapenem for three weeks. It has been one year since his last episode now, and he continues to follow up with the ID clinic for the management of his HIV symptoms.

## Discussion

This case of recurrent MDR shigellosis in a young man with advanced HIV and high-risk sexual behaviors highlights the complex interplay of individual choices, immune suppression, AMR, and the healthcare system. His repeated engagement in unprotected sexual activity and suboptimal HIV management likely facilitated both recurrent exposure to *Shigella* and the selection of MDR strains.

This case aligns with the growing recognition of shigellosis as a sexually transmitted infection among MSM, a trend observed since outbreaks were first documented in San Francisco in 1974 [5]. Subsequent outbreaks in major cities worldwide, including Seattle, Vancouver, Sydney, London, Paris, and Tokyo, underscore the global reach and persistence of this issue within the MSM community [6-8]. The phenomenon of serological sorting, as described by Aragón et al., where individuals with HIV seek partners with similar serostatus, may further concentrate resistant strains within this population [6].

This patient's experience highlights several critical challenges. Inadequate HIV treatment not only weakens the immune system, making individuals more susceptible to shigellosis, but also potentially allows for prolonged *Shigella* shedding and the development of resistance [7]. The patient's failure to recognize the severity of his condition, adhere to treatment, and attend follow-up appointments reveals the need for targeted patient education and engagement strategies. Additionally, gaps in healthcare coordination between hospitalizations and outpatient care likely contributed to treatment failures and recurrent infections. Underreporting of shigellosis cases can further hinder surveillance efforts and delay public health responses, particularly in vulnerable populations.

Although most cases of shigellosis are self-limiting or resolved with supportive care (e.g., dehydration management), antibiotics are suggested for those with sequelae or long-term symptoms. The WHO recommends ciprofloxacin as a first-line treatment for shigellosis and azithromycin, ceftriaxone, or pivmecillinam as second-line medicines [7]. The elevated risk of AMR in high-risk MSM (hrMSM) can be attributed to two key factors. First, compared to heterosexuals, hrMSM engages in a higher prevalence of oro-penile, oro-rectal, and anal sex, which leads to increased mixing of microbes from the pharyngeal, rectal, and penile microbiomes. Second, hrMSM tends to have a greater number of sexual partners and overlapping sexual relationships, further facilitating the exchange of microbes. This heightened exchange of microbiomes across different body habitats, combined with the interconnectedness of sexual networks in this population, creates a unique ecosystem that fosters the development and spread of antibiotic resistance [8].

The emergence of MDR shigellosis in MSM with HIV represents a "perfect storm" of factors, including behavioral co-factors (e.g., prevention fatigue, substance use), biological susceptibility due to HIV, and the dynamics of sexual networks that facilitate transmission [9]. The escalating prevalence of resistance, evident in NARMS data since 1999, further emphasizes the urgency of addressing this issue. The evolution of resistance to traditional treatments like sulfonamides, tetracycline, ampicillin, and even quinolones necessitates ongoing research into novel therapeutic approaches [10].

## Conclusions

This case of recurrent MDR shigellosis in a young man with advanced HIV underscores the complex interplay of factors contributing to this emerging sexually transmitted infection. His repeated infections despite multiple courses of antibiotics highlight the critical need for comprehensive HIV care, including early diagnosis, initiation of effective antiretroviral therapy, and adherence support to bolster immune function. Furthermore, extensive patient education regarding safe sexual practices and the importance of treatment adherence is crucial to prevent recurrent infections and transmission. Robust healthcare coordination is essential to bridge gaps in care and promote optimal patient outcomes. Finally, strengthened surveillance and reporting mechanisms are vital to inform public health interventions, guide antibiotic stewardship efforts, and ultimately curb the spread of multidrug-resistant shigellosis within vulnerable populations. This case serves as a stark reminder of the multifaceted challenges in managing this emerging infection. It emphasizes the need for a comprehensive, collaborative approach to effectively combat this public health threat. Given the persistent outbreaks of shigellosis among MSM, healthcare providers should consider recommending that this population avoid direct oral-anal sexual contact, particularly during periods of illness or community outbreaks.
